# Selective antimicrobial photodynamic therapy of clinical isolates from patients with infected diabetic foot ulcers using a small cationic chlorin

**DOI:** 10.1128/aac.00962-25

**Published:** 2025-11-18

**Authors:** Anita S. Amorim, Chloe M. Hobbs, Zoe A. Arnaut, Mariette M. Pereira, David Gallagher, Teck Wee Boo, Georgina Gethin, James P. O'Gara, Luis G. Arnaut

**Affiliations:** 1 Chemistry Department, CQC-IMS, University of Coimbra 37829, Coimbra, Portugal; 2 Microbiology, School of Biological and Chemical Sciences, University of Galway 8799, Galway, Ireland; 3 School of Medicine, University of Galway 162643, Galway, Ireland; 4 School of Nursing and Midwifery, University of Galway 111805, Galway, Ireland; University of Pennsylvania Perelman School of Medicine, Philadelphia, Pennsylvania, USA

**Keywords:** diabetic foot ulcers, photodynamic inactivation, antimicrobial resistance, biofilms, infection

## Abstract

Diabetic foot ulcers (DFUs) are a major complication of diabetes mellitus and the principal factor contributing to lower limb amputations. Antimicrobial photodynamic therapy (aPDT) is a promising therapeutic strategy because it is not limited by drug resistance. A new photosensitizer, a low molecular weight dicationic imidazolyl chlorin (IC-H-Me^2+^), was developed for aPDT to improve permeation in biofilms and light absorption in the phototherapeutic window. The efficacy of aPDT with IC-H-Me^2+^ was tested against biofilms produced by methicillin-resistant *Staphylococcus aureus* (MRSA), methicillin-susceptible *S. aureus* (MSSA), *Staphylococcus epidermidis,* and *Pseudomonas aeruginosa* isolates from patients with DFUs, both alone and combined with potassium iodide (KI). aPDT with 1 µM IC-H-Me^2+^ and a 1-h incubation period, followed by exposure to a light dose of 5 J/cm^2^ using a 660 nm LED, reduced the viability of all biofilms by >3 log of colony-forming units (CFU) in one single aPDT session. Furthermore, when this photosensitizer and light doses were combined with 50 mM KI, most biofilms were completely eradicated. This combination generates triiodide ions in the biofilms, which increase the response to aPDT. Up to 15 log CFU/mL reduction of biofilms was obtained with one single treatment. This same combination does not significantly reduce the viability of human epidermal keratinocytes. The eradication of MRSA biofilms from clinical isolates is selective. These results demonstrate that IC-H-Me^2+^, properly formulated with KI, has the potential to selectively eradicate biofilms established in infected wounds and decrease amputation rates in patients with DFU.

## INTRODUCTION

Diabetic foot ulcers occur in 15% of patients with diabetes mellitus due to factors such as neuropathy, repeated trauma, and peripheral vascular disease (1). This represents a tremendous disease burden because the global diabetes prevalence in 20–79 year olds exceeds 10% (2). Diabetic foot ulcers (DFUs) are generally colonized by complex polymicrobial communities. However, Gram-positive cocci, including *Staphylococcus aureus*, are among the most frequently identified bacteria (3). *S. aureus* infections of chronic wounds are particularly problematic due to the ability of the pathogen to produce biofilm, resulting in complex and difficult-to-treat infections within the tissue and underlying bone (4, 5). Furthermore, *S. aureus* has been implicated as a causative pathogen in approximately 60% of osteomyelitis cases (4).

A prospective observational study showed that, within the first year after presentation with a diabetic foot infection (DFI), the morbidity rate reached 15%, 17% of patients had undergone a lower extremity amputation, 10% had recurrences, and resolution of the ulcer occurred in only 45% (6). The latest guidelines on the treatment of DFIs caution against topical antiseptics, silver preparations, honey, bacteriophage therapy, or negative pressure wound therapy (7). Antiseptics containing iodine seem to be safe and may achieve full closure of the wound, but they require months of regular treatment (8). The clinical outcomes of DFIs are generally poor.

An underexplored option to inactivate multidrug-resistant bacteria is antimicrobial photodynamic therapy (aPDT) (9). PDT employs a photosensitizer that absorbs light and interacts with oxygen to generate reactive oxygen species (ROS), such as singlet oxygen, superoxide ion, hydrogen peroxide, or hydroxyl radicals. The low penetration of light in tissues is often cited as a limitation for PDT, but bacteria are not normally seen at a depth more than 1.5 mm from the surface of the wound (10), and the optical penetration depth of skin for wavelengths above 600 nm is higher than 1.5 mm (11). A more serious limitation of aPDT is selectivity toward bacteria. The ROS generated by PDT causes oxidative stress that can kill both bacterial and mammalian cells (12). Host cells can be spared by directing light to the infected tissue because ROS action is local, but it is very challenging to find a regimen where the biofilm of an infected wound is eradicated without damaging host tissues and impairing healing. A good aPDT protocol should be able to inactivate >99.9% of prokaryotic cells in biofilms while sparing >70% of eukaryotic cells.

A photosensitizer based on the phthalocyanine structure (RLP068) showed selectivity toward planktonic bacteria at short incubation times (13) and did not generate resistant mutants after 20 consecutive aPDT treatments (14), but biofilms typically require orders of magnitude higher doses to be destroyed (9). Nevertheless, the DANTE clinical study showed good tolerability to aPDT with 0.30% RLP068 and a transient decrease of microbial load by ~3 logarithms of colony-forming units (CFU) per milliliter (15). Repeated treatments of DFI with RLP068-aPDT twice a week for 1 month reduced treatment times and antibiotics use and the incidence of amputations (16). We have previously reported on the selective eradication of planktonic *S. aureus* and *Escherichia coli* with a small dicationic chlorin (IC-H-Me^2+^) using a light dose of 5 J cm^–2^ at 660 nm delivered after 1 h of incubation (17). The eradication of biofilms required the potentiation of 1 µM IC-H-Me^2+^ with 50 mM potassium iodide, under the same illumination conditions, which allows for >70% viability of mammalian cells (18).

In this work, we investigate the photo-inactivation of biofilms collected from patients with DFI using IC-H-Me^2+^ and KI. Fourteen clinical isolates collected from the wounds of 12 patients were grown to form mature biofilms. They contained methicillin-susceptible *S. aureus* (MSSA), methicillin-resistant *S. aureus* (MRSA), *Staphylococcus epidermidis*, *Pseudomonas aeruginosa*, *Staphylococcus simulans,* and *Staphylococcus caprae*. These isolates include the bacterial species most frequently found in DFUs. An aPDT protocol known to spare >70% of mammalian cells *in vitro* decreased the viability of these biofilms by unprecedented >7 log CFU/mL after one single treatment.

## RESULTS

### DFI clinical isolates produce polysaccharide and protein adhesin-mediated biofilms

**Fig 1:**
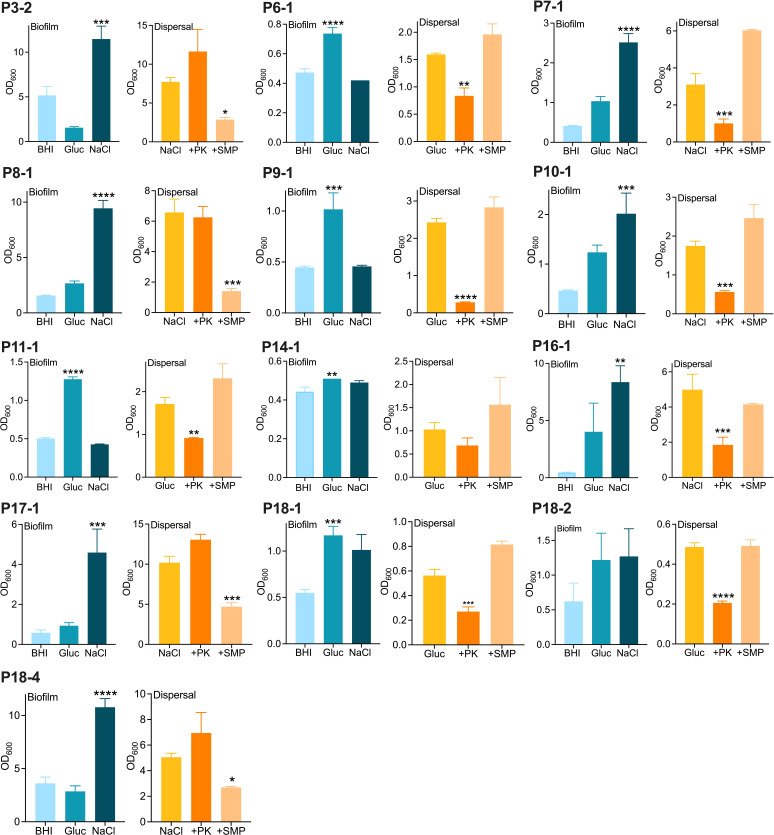
Staphylococcal isolates from patients with DFIs produce polysaccharide or protein adhesin-mediated biofilms. Left-hand side of each panel: biofilms grown at 37°C for 24 h in BHI broth, BHI glucose, and BHI NaCl. Right-hand side of each panel: biofilms grown for 24 h in the conditions that induce the highest level of biofilm were left untreated or dispersed by proteinase K (PK) or sodium metaperiodate (SMP) for a further 24 h. The data are the average of three biological repeats, and standard deviations are shown. Statistical significance was determined using one-way analysis of variance (ANOVA) in GraphPad Prism (*P < 0.05, ** *P* < 0.01, *** *P* < 0.001, and **** *P* < 0.0001).

Table 1 briefly describes the DFI clinical isolates used in this study. Biofilm production by these isolates was compared in brain-heart infusion (BHI), BHI glucose, and BHI NaCl. Osmotic stress induced by NaCl was previously shown to activate the *icaADBC* operon, resulting in increased production of PIA/PNAG type biofilms (19, 20), whereas growth in BHI glucose is associated with the production of fibronectin-binding protein-mediated biofilm (21). *Staphylococcus epidermidis* isolates P3-2 and P18-4, and MSSA isolates P7-1, P8-1, P10-1, P16-1, and P17-1 all produced significantly more biofilm in BHI NaCl (Fig. 1). MSSA isolates P6-1, P9-1, MRSA isolate P11-1, and *S. simulans* P18-1 produced significantly more biofilm in BHI glucose. Biofilm production by MSSA P14-1 was not significantly induced by NaCl or glucose, while *S. caprae* P18-2 biofilm production was similar under all growth conditions. *P. aeruginosa* is known to predominantly produce polysaccharide matrix biofilms (22), and its biofilm matrix was not analyzed in this work.

**TABLE 1:** Clinical isolates from patients with diabetic foot infections used in this study

Isolate identification code	Isolate type	Sample collection method	Wound duration	Comorbidities^*a*^
P3-2	*Staphylococcus epidermidis*	Tissue biopsy	6 weeks	T2DM
P6-1	Methicillin-susceptible *Staphylococcus* *aureus* (MSSA)	Tissue biopsy	–^*b*^	T2DM, chronic kidney disease, atrial fibrillation
P7-1	MSSA	Tissue biopsy	8 weeks	T2DM, neuropathy, osteomyelitis
P8-1	MSSA	Tissue biopsy/blood cultures	10 weeks	T2DM, PVD
P9-1	MSSA	Tissue biopsy	3 weeks	T2DM, PVD
P10-1	MSSA	Wound swab	12 weeks	T2DM, neuropathy, obesity, Charcot arthropathy
P11-1	Methicillin-resistant *Staphylococcus aureus* (MRSA)	Wound swab	8 weeks	T1DM, PVD
P12-1	*Pseudomonas aeruginosa*	Wound swab		Late onset T1DM, neuropathy
P14-1	MSSA	Bone biopsy	9 months	T2DM, rheumatoid arthritis
P16-1	MSSA	Bone biopsy	–	–
P17-1	MSSA	Wound swab	–	–
P18-1	*Staphylococcus simulans*	Tissue biopsy	–	–
P18-2	*Staphylococcus caprae*	Tissue biopsy	–	–
P18-4	*Staphylococcus epidermidis*	Tissue biopsy	–	–

^*a*^ T2DM, type 2 diabetes mellitus; T1DM, type 1 diabetes mellitus; PVD, 119 peripheral vascular disease.

^*b*^ “–” indicates unknown wound duration.

Biofilm dispersal assays with sodium metaperiodate (SMP) and proteinase K (PK) revealed roles for polysaccharide adhesin in isolates P3-2, P18-4, P17-1, and P8-1 and protein adhesins in the remaining isolates except P14-1 (Fig. 1). Notably, biofilm induction by NaCl or BHI glucose alone was not a reliable indicator of biofilm matrix composition and needed to be supported by dispersal assays. Five isolates, P3-2, P8-1, P16-1, P17-1, and P18-4, were classified as hyper biofilm producers (OD_600_ > 5) in 24 and 48 h biofilm assays. Among these, P3-2, P8-1, P17-1, and P18-4 produced predominantly polysaccharide matrix biofilms, whereas P16-1 produced predominantly protein adhesin-type biofilm.

### aPDT combined with KI in nontoxic conditions can inactivate 15 log CFU/mL of biofilms formed by clinical isolates

**Fig 2:**
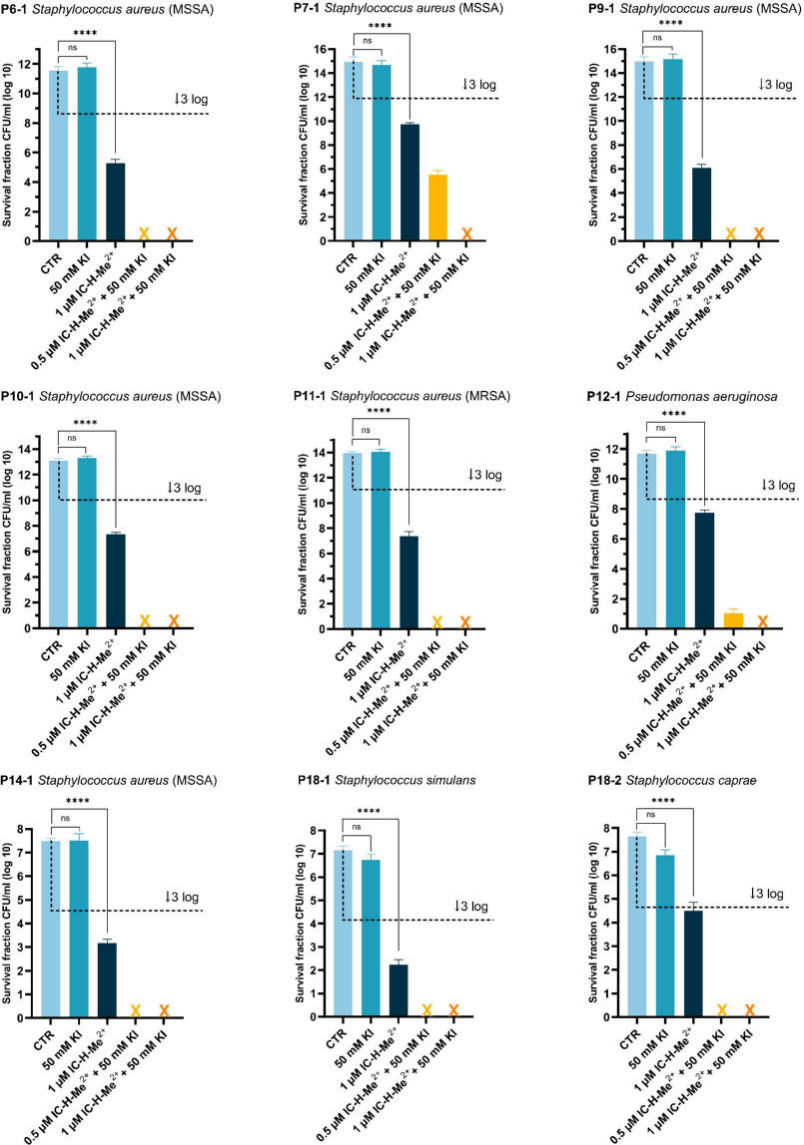
Photodynamic inactivation of moderate biofilm-producing isolates P6-1, P9-1, P11-1, P14-1, P18-1, and P18-2 biofilms grown in BHI glucose, and P7-1, P10-1, and P12-1 in BHI NaCl at 37°C for 24 h, at 5 J cm^−2^ with 1 µM PS alone or with combinations of 0.5 or 1 µM PS with 50 mM KI. CTR represents untreated biofilms (no photosensitizer, no light). A control with 50 mM KI is also presented. Biofilm viability was determined by enumerating CFUs after aPDT. The dashed black line indicates 99.9% (3 log) inactivation of bacterial cells, and the crosses indicate complete inactivation of the bacteria below the detection limit of 10 CFUs. The data are expressed as the mean of three independent experiments (*n* = 3) ± the standard error of the mean (SEM). Statistical significance was determined using one-way ANOVA (ns, not significant; **** *P* < 0.0001).

aPDT of biofilms produced by clinical isolates was first tested with 1 µM IC-H-Me^2+^ alone and with 0.5 or 1 µM IC-H-Me^2+^ combined with 50 mM KI. For the moderate biofilm-producing isolates, the aPDT regime with 1 µM IC-H-Me^2+^ alone achieved ≥3 log CFU/mL reductions in biofilm viability (Fig. 2). Furthermore, the addition of 50 mM KI in the aPDT regime led to a complete inactivation of biofilms produced by all of these isolates (Fig. 2). This complete inactivation of clinical biofilms with one aPDT session under conditions where the viability of HaCaT (human epidermal keratinocytes) cells is higher than 80% is unprecedented (9).

**Fig 3:**
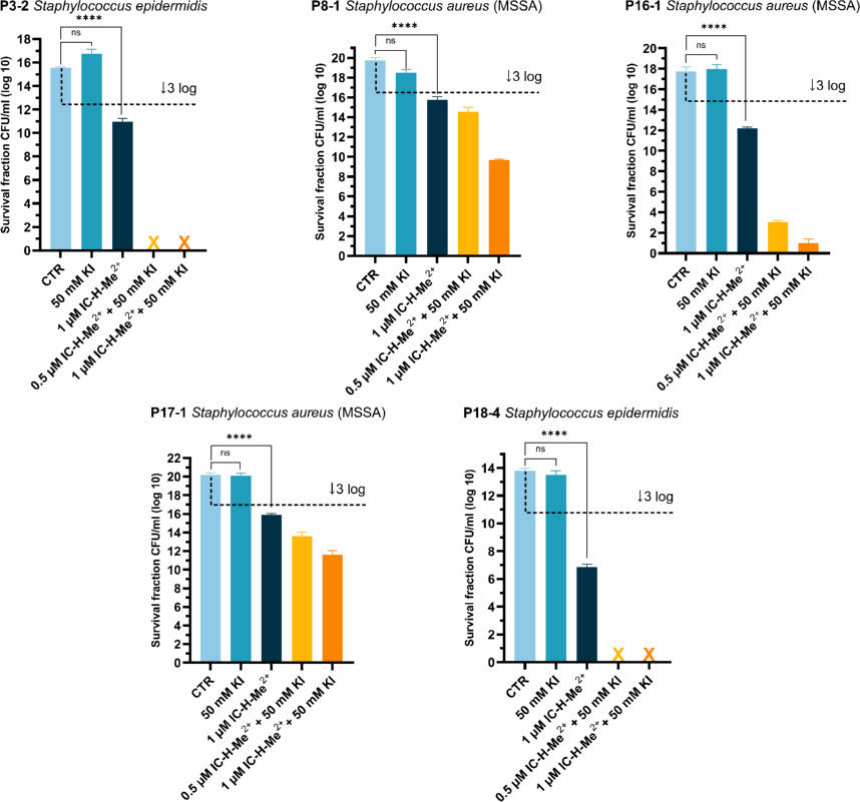
Photodynamic inactivation of hyper biofilm-producing isolates P3-2, P8-1, P16-1, P17-1, and P18-4 biofilms grown in BHI NaCl at 37°C for 24 h, at 5 J cm^−2^ with 1 µM PS alone or with combinations of 0.5 or 1 µM PS with 50 mM KI. CTR represents untreated biofilms. A control with 50 mM KI is also presented. Biofilm viability was determined by enumerating CFUs after aPDT. The dashed black line indicates 99.9% (3 log) inactivation of bacterial cells, and the crosses indicate complete inactivation of the bacteria below the detection limit of 10 CFUs. The data are expressed as the mean of three independent experiments (*n* = 3) ± SEM. Statistical significance was determined using one-way ANOVA (ns, not significant; **** *P* < 0.0001).

This aPDT regime also significantly inactivated biofilms produced by the hyper biofilm-producing staphylococcal strains but did not achieve a complete inactivation of P8-1, P16-1, and P17-1 biofilms (Fig. 3). These data indicate that the capacity for hyper biofilm production does not necessarily imply that aPDT will be less effective. Among the five hyper biofilm-forming isolates, not all showed high-level tolerance to aPDT. P8-1 and P17-1, and to a lesser extent P16-1, which had the highest biofilm CFU densities (>15 log CFU/mL), were able to survive this aPDT regime, whereas P3-2 and P18-4 were completely eradicated. These data also indicate that biofilm matrix type does not correlate with susceptibility to aPDT. P3-2, P8-1, P17-1, and P18-4 all produce predominantly polysaccharide matrix-type biofilms, but only P8-1 and P17-1 exhibit higher-level tolerance to aPDT. Finally, all isolates that produce protein adhesin-type biofilms, including MRSA isolate P11-1 and hyper biofilm producer P16-1, were highly susceptible to this aPDT regimen.

As with the staphylococcal isolates, the viability of *P. aeruginosa* P12-1 biofilms was reduced by >3 log with 1 µM IC-H-Me^2+^ alone and completely inactivated by 1 µM IC-H-Me^2+^ and 50 mM KI (Fig. 2), indicating that this aPDT regime may be active against diverse Gram-positive and Gram-negative pathogens. This is consistent with other studies using cationic photosensitizers (18, 23–25).

### aPDT with KI generates I_3_^−^ in the presence of 4% NaCl or 1% glucose

**Fig 4:**
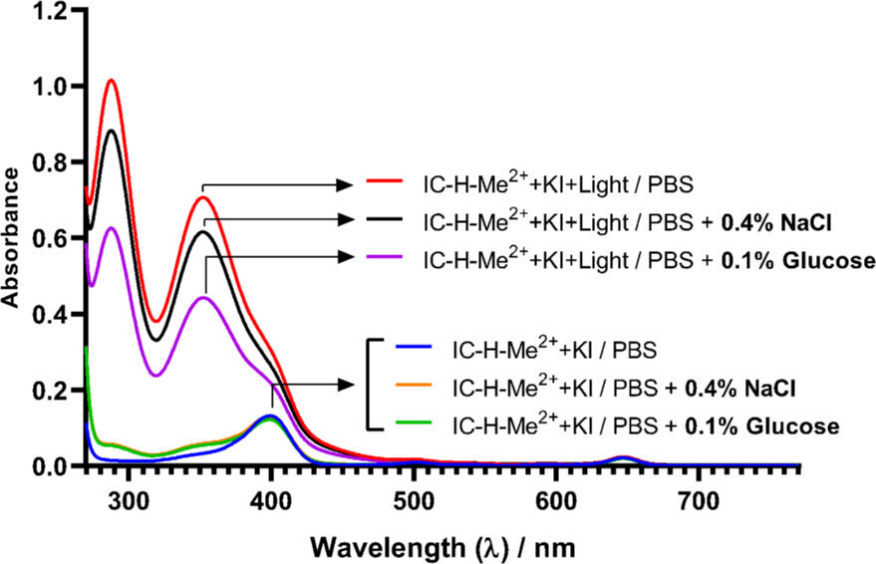
Absorption spectra in PBS, 0.4% NaCl, or 0.1% glucose, of 1 µM of IC-H-Me^2+^ with 50 mM KI, before and after illumination with 5 J cm^−2^ at 660 nm.

The potentiation of aPDT with KI is mediated by the formation of I_3_^−^ (18, 26). We investigated the formation of I_3_^−^ in the presence of NaCl and glucose to assess their impact on the potentiation mechanism. Figure 4 shows that, prior to illumination, the absorption spectrum of the medium with IC-H-Me^2+^ and KI is dominated by the characteristic bands of the chlorin at ~400 and ~650 nm. After illumination, the spectrum is dominated by intense bands at 290 and 350 nm, assigned to I_3_^−^ (18). Considering that the biofilms are washed once with phosphate-buffered saline (PBS) before incubation with IC-H-Me^2+^ and KI, I_3_^−^ photogeneration studies employed 10% of the concentration of NaCl and glucose in the growth medium.

The presence of NaCl does not appreciably affect the photogeneration of I_3_^−^ and does not explain the higher tolerance of P8-1 and P17-1 to aPDT. Interestingly, the absorption of photogenerated I_3_^−^ decreases in 0.1% glucose. The addition of I_3_^−^ to polysaccharides produces the starch-iodine complex blue color. Adding I_3_^−^ to small chain length polysaccharides does not change the I_3_^−^ bands’ positions but increases their intensity (27). This further suggests that glucose impairs the formation of I_3_^−^. The photochemical generation of I_3_^−^ involves singlet oxygen, which is known to react with glucose (28). This suggests that while the biofilm response to aPDT may be lower in BHI glucose, this is not a predictor of aPDT effectiveness, which, as noted above, appears to be a function of biofilm production and biofilm cell density.

### Glucose increases the tolerance of hyper biofilm producers to aPDT, and trypsin increases their susceptibility

**Fig 5:**
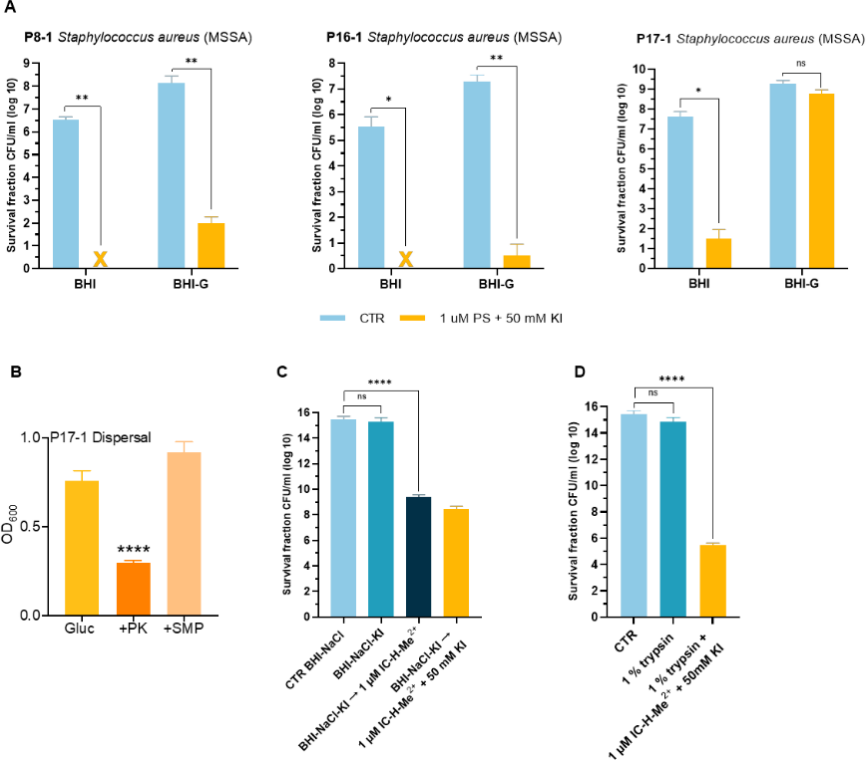
Environmental effects on the response of hyper biofilm producers to aPDT. (**A**) P8-1, P16-1, and P17-1 biofilms grown in BHI or BHI glucose at 37°C for 24 h before aPDT (5 J/cm^2^) with 1 µM PS and 50 mM KI. (**B**) P17-1 biofilms grown for 24 h in BHI glucose were left untreated or dispersed by PK or SMP for a further 24 h. (**C**) P17-1 biofilms grown in BHI NaCl or in BHI NaCl KI at 37°C for 24 h before aPDT (5 J/cm^2^) with 1 µM PS and 50 mM KI. (**D**) P17-1 biofilms grown in BHI NaCl at 37°C for 24 h before aPDT (5 J/cm^2^) with 1 µM PS, 50 mM KI, and trypsin. Biofilm viability was determined by enumerating CFUs after aPDT. The crosses indicate complete inactivation of the bacteria below the detection limit of 10 CFUs. The data are expressed as the mean of three independent experiments (*n* = 3) ± SEM. Statistical significance was determined using one-way ANOVA (ns, not significant; * *P* < 0.05, ** *P* < 0.01, and **** *P* < 0.0001).

P8-1, P16-1, and P17-1 biofilms grown in BHI NaCl exhibited the highest level of tolerance to aPDT (Fig. 3). Biofilm production by all three isolates was also significantly increased in BHI glucose, albeit not to the same extent as BHI NaCl (Fig. 1). Figure 5 shows that aPDT can eradicate P8-1 and P16-1 biofilms grown in BHI but not in BHI glucose. Moreover, P17-1 biofilms grown in BHI glucose were not significantly inactivated by aPDT (Fig. 5A), whereas aPDT led to a significant >3 log reduction in the viability of P17-1 biofilms grown in BHI NaCl (Fig. 3). This higher tolerance in BHI glucose may be related to the decreased photochemical generation of I_3_^−^ in the presence of glucose, discussed above, or to altered matrix composition in BHI glucose. Consistent with the latter possibility, P17-1 biofilms grown in BHI glucose were significantly dispersed with proteinase K (Fig. 5B), indicating that this isolate may utilize both polysaccharide and protein adhesins to produce biofilm.

We further attempted to enhance the eradication of P17-1 biofilms grown in BHI NaCl by increasing the KI incubation time to 24 h, with the expectation that prolonged incubation improves I_3_^−^ infiltration in the biofilm. This procedure resulted in a marginal increase in aPDT efficacy (Fig. 5C). Biofilm washing before incubation with IC-H-Me^2+^ likely removes most I_3_^−^ and suggests that its infiltration in the biofilm is not limiting the response to aPDT.

Finally, we investigated if the debridement agent trypsin might improve P17-1 biofilm susceptibility to aPDT. Trypsin is a digestive protease widely used in chronic wound care (29). Figure 5D shows that 10 min incubation with 1% trypsin has no effect on the viability of P17-1 biofilms, but when combined with aPDT, it reduces the viability of bacteria by 10 log CFU/mL, i.e., 2 log CFU/mL more than aPDT in the absence of trypsin. Trypsin incubation facilitates the association of IC-H-Me^2+^ with bacteria and improves the response of P17-1 biofilms. Although this hyper biofilm producer has a predominantly polysaccharide matrix, the 10 log CFU/mL reduction is not sufficient to eradicate P17-1 biofilms.

### Confocal microscopy shows that hyper biofilm producers impair the association of the photosensitizer with the bacteria

**Fig 6:**
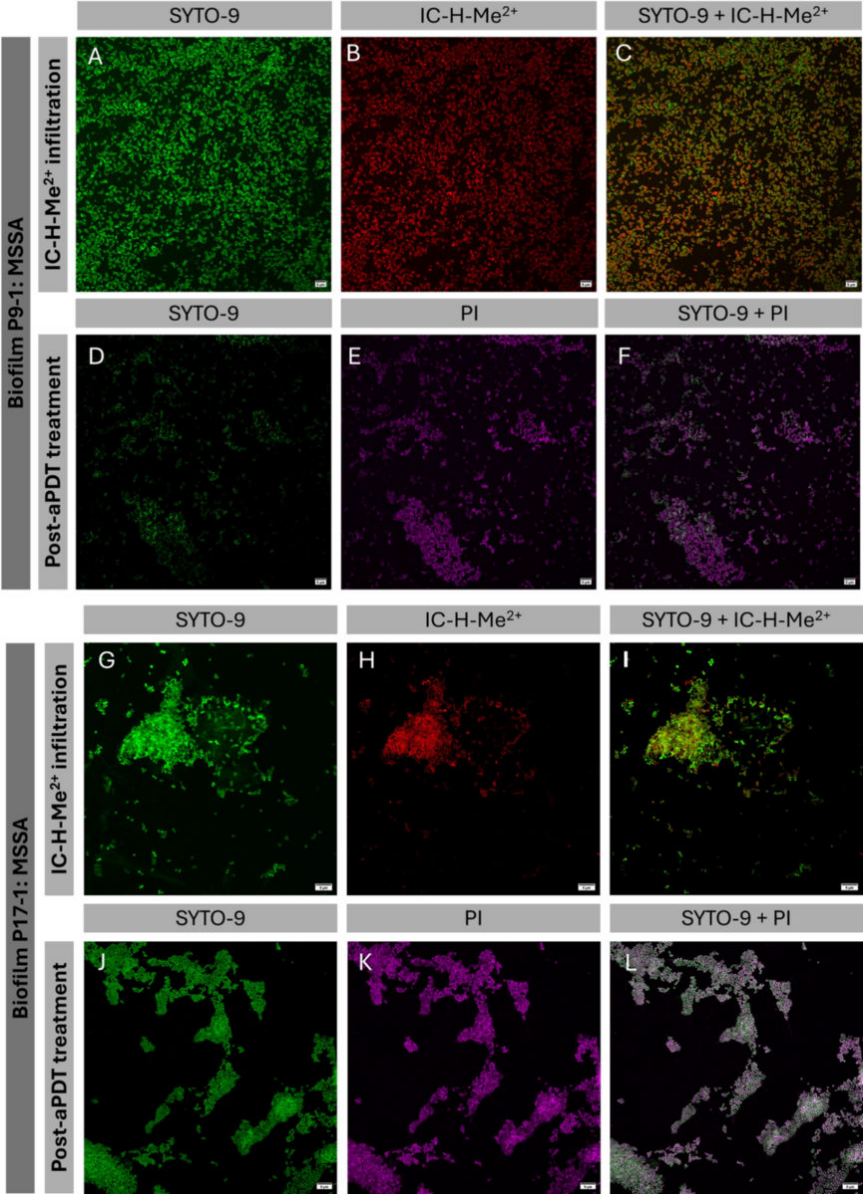
Confocal microscopy of MSSA isolates. The P9-1 biofilm of protein matrix was grown in BHI glucose (**A and F**), and the P17-1 biofilm of polysaccharide matrix was grown in BHI NaCl (**G and L**). (**A–C**) and (**G–I**) were obtained before aPDT with 5 J cm^−2^, 1 µM IC-H-Me^2+^, and 50 mM KI, whereas (**D–F**) and (**J–L**) were collected after aPDT. Biofilms were stained with SYTO-9 (green color) and PI (pink color), and incubated with IC-H-Me^2+^ (red color). Selective imaging employed for SYTO-9 λ_exc_ = 488 nm/λ_em_ = 500–540 nm (**A, D, G, J**), for IC-H-Me^2+^ 405 nm/660–760 nm (**B and H**), and for PI 561 nm/500–540 nm (**E and K**). (**C and I**) Inform on the co-localization of all bacteria and photosensitizers. (**F and L**) Inform on co-localization of all bacteria and dead bacteria.

Green-fluorescent SYTO-9 binds DNA and RNA in both live and dead Gram-positive and Gram-negative bacteria (30). Combining SYTO-9 with propidium iodide (PI), which only permeates the cell envelope of dead bacteria and emits orange/red fluorescence, allows for the differentiation between live and dead bacteria. IC-H-Me^2+^ has a distinct red/near-infrared fluorescence. Confocal microscopy can use these distinct fluorescences to visualize simultaneously live and dead bacteria and photosensitizer localization (Fig. 6).

Figure 6C shows that IC-H-Me^2+^ (red) is superposed with bacterial cells (green). In contrast, Fig. 6I shows regions of intense green color that do not overlap with red, i.e., some of the bacteria are not associated with the photosensitizer. Figure 6F, obtained after aPDT, shows virtually no bacteria that are not simultaneously stained by SYTO-9 and PI, meaning that nearly all bacteria are dead. In contrast, Fig. 6L shows cell clusters with predominantly green color, from which KI is absent, which means that most of the bacteria are alive. These data indicate that biofilm eradication with aPDT depends on the association of IC-H-Me^2+^ with the bacterial cells.

## DISCUSSION

Our main goal was to assess the feasibility of treating DFI with aPDT combining IC-H-Me^2+^ with KI under conditions that are not toxic to human cells. Clinical isolates from 12 patients were grown to mature biofilms and treated once with aPDT to make this assessment.

Biofilm characterization revealed that DFI isolates P3-2 (*S. epidermidis*), P8-1 (MSSA), P17-1 (MSSA), and P18-4 (*S. epidermidis*) were significantly eradicated when treated with sodium metaperiodate but not with proteinase K, indicating that these isolates produce a polysaccharide-type biofilm *in vitro* as opposed to an adhesin-type biofilm. On the contrary, biofilms produced by P6-1 (MSSA), P7-1 (MSSA), P9-1 (MSSA), P10-1 (MSSA), P11-1 (MRSA), P14-1 (MSSA), P16-1 (MSSA), P18-1 (*S. simulans*), and P18-2 (*S. caprae*) were all significantly eradicated when treated with proteinase K, indicating the production of a protein-type biofilm.

IC-H-Me^2+^ has a strong absorption band at 651 nm that is very convenient for aPDT (17). Previously, 1 µM IC-H-Me^2+^ combined with 50 mM KI illuminated for 1 h with 5 J/cm^2^ at 660 nm was shown to eradicate *S. aureus* ATCC 292131 or *Escherichia coli* ATCC 25922 biofilms (7 to 11 log CFU/mL reduction) without significantly affecting the viability of human epidermal keratinocyte (HaCaT) cells (>70% survival) (18). The same protocol (1 µM IC-H-Me^2+^, 50 mM KI, 1 h incubation, 5 J/cm^2^ at 660 nm) enabled up to 15 log CFU/mL reduction of biofilms grown from DFI isolates. The excellent response to aPDT was also observed with the Gram-negative bacterium *P. aeruginosa*, isolated from the wound site of a patient who had recently undergone a minor lower extremity amputation as a result of a DFI (~4 log CFU/mL reduction with 1 µM IC-H-Me^2+^ and 12 log CFU/mL reduction when combined with 50 mM KI).

This success with biofilms formed by Gram-negative bacteria is especially remarkable because other photosensitizers require concentrations higher than 30 µM to obtain a bactericidal effect (>3 log CFU/mL reduction) in such biofilms, and these high concentrations compromise selectivity (9). The combination with KI improves the response to aPDT for various, but not all, photosensitizers. For example, an 8 log CFU/mL reduction of *P. aeruginosa* biofilms was obtained with 20 µM methylene blue combined with 100 mM KI and 60 J cm^−2^, but the combination of curcumin with KI was unsuccessful (31). One of the most impressive combinations of photosensitizers with 100 µM KI is that of FORM, a non-separated mixture of five cationic *meso*-tetraarylporphyrins, which reduced the load of Gram-positive and Gram-negative biofilms by 7.5 log CFU/mL at 1 µM concentration (32). The log CFU/mL reductions attained with the combination of IC-H-Me^2+^ with KI are much higher than those previously reported, even for the case of the five hyper-biofilm-forming isolates presented in this work. Three of these isolates grew to 18 to 20 log CFU/mL after 24 h incubation. The viabilities of these extremely high cell density biofilms were significantly reduced (>9 log CFU/mL), but not fully eradicated, with one aPDT session.

Studies of the hyper-biofilm-forming isolates that were less susceptible to aPDT (P8-1, P16-1, and P17-1) revealed that their tolerance was not related to the BHI NaCl medium. Two of them (P8-1 and P17-1) produce predominantly polysaccharide matrix-type biofilms, whereas the other (P16-1) produces protein adhesin-type biofilms. P17-1 exhibited the highest-level tolerance to aPDT, particularly under biofilm-inducing growth conditions in BHI glucose. Predominantly polysaccharide matrix-type biofilms proved to be more difficult to inactivate with aPDT, but the major factor contributing to tolerance to PDT was the dense aggregation of bacteria produced by hyper-biofilm strains. This does not seem to impair the diffusion of I^−^ into the biofilms, but does limit the penetration of IC-H-Me^2+^. Confocal microscopy of hyper-biofilm-forming isolates reveals that IC-H-Me^2+^ was not distributed evenly throughout the biofilm. Bacteria in biofilm regions with low IC-H-Me^2+^ concentrations were better able to survive aPDT and avoided complete eradication. Nevertheless, this limitation should not obscure the fact that hyper-biofilm-forming isolates with less than 16 log CFU/mL were eradicated with just one aPDT session.

In conclusion, using a protocol known to maintain the viability of >70% of mammalian cells, we demonstrated the complete eradication or significant reduction in the viability of biofilms produced by clinical isolates from patients with DFIs after one single aPDT session with IC-H-Me^2+^ combined with KI. Gram-positive and Gram-negative bacteria responded equally well. MRSA was as susceptible to aPDT as MSSA. aPDT with IC-H-Me^2+^ combined with KI has the potential to improve the management of patients with DFIs.

## MATERIALS AND METHODS

### Chemicals and reagents

Potassium iodide, SMP, and PK were purchased from Sigma-Aldrich. The photosensitizer 5,15-bis(1,3-dimethylimidazol-2-yl) chlorin diiodide (IC-H-Me^2+^) was prepared as previously reported (17). IC-H-Me^2+^ stock solutions were prepared in PBS and diluted in PBS to obtain the desired concentrations. The KI stock solution was prepared in PBS.

### Clinical isolates and ethics approval

Enrolled patients were over 18 years, diagnosed with an active DFI, and were not receiving antibiotics at the time of sample collection. All patient data collected was anonymized and linked only to the patient number allocated for the purpose of this study.

Skin and soft tissue biopsies were the preferred method of specimen collection. Bone biopsies were collected from patients with suspected osteomyelitis. Where biopsies were not possible or deemed unsuitable by healthcare providers, swabs were taken from the wound surface. All wounds were prepared using sharp debridement and cleansing to remove wound slough and exudate prior to specimen collection. All specimens were collected as part of the routine care provided to the patients, and not solely for the purpose of this study. Microorganisms present in the specimens were cultured and identified in the Bacteriology department of University Hospital Galway.

### Biofilm formation assays

Biofilms were grown in 96-well Nunclon tissue culture-coated microtiter plates in BHI or BHI supplemented with 1% glucose (BHI glucose) or 4% NaCl (BHI NaCl) as described previously (5, 19–21). Briefly, biofilms were grown in 200 µL cultures for 24 h at 37°C in 96-well plates before being washed three times by fully submerging the plates in water. Biofilm biomass was fixed at 60°C for 1 h before being stained with 0.1% crystal violet. A 20% acetic acid solution was used to solubilize crystal violet associated with the biofilms, and the absorbance was measured at OD_600_ using a TECAN plate reader. OD_600_ readings in the linear region were obtained by dilution as required.

### Biofilm dispersal assays

Biofilms were grown for 24 h in BHI glucose or BHI NaCl, depending on which growth condition induced the highest level of biofilm for each strain. Next, 40 mM SMP or 1 mg/mL PK was added directly to the growth media, and the plates were re-incubated for a further 24 h at 37°C. For the untreated controls, biofilms were grown in BHI glucose or BHI NaCl for 48 h. Thereafter, the supernatant was removed and the plates washed three times via submersion in water, before being stained with a 0.1% crystal violet solution and the biofilm quantified as described above.

### aPDT of biofilms

Biofilms grown as described above were washed with PBS and incubated with 0.5 or 1 µM IC-H-Me^2+^ and, in the conditions identified in Fig. 2, 3, and 5, with the label “+50 mM KI,” also with 50 mM KI, for 60 min in the dark at room temperature. Controls were incubated with PBS only. Next, the plates were irradiated with 660 nm LEDs with a light dose of 5 J/cm^2^. After illumination, the biofilms were carefully scraped and sonicated before CFUs were enumerated on Mueller-Hinton agar plates incubated for 24 h at 37°C in the dark.

### Environmental effects on aPDT

Biofilms were grown from the P17 isolate for 24 h in BHI NaCl (control) or BHI NaCl plus 50 mM KI before being washed and incubated with 1 µM IC-H-Me^2+^ and 50 mM KI for 60 min in the dark at room temperature and then irradiated with 660 nm LED (5 J/cm^2^). After irradiation, biofilm survival was determined by enumerating CFUs as described above.

Biofilms grown for 24 h was first incubated in 1% trypsin for 10 min, before being washed twice, and aPDT was performed as described above with 1 µM IC-H-Me^2+^ and 50 mM KI.

### Formation of the triiodide species (I_3_^−^)

Illumination with 5 J/cm^2^ at 660 nm of 1 µM IC-H-Me^2+^ in the presence of 50 mM KI was performed in PBS with 0.1% glucose or with 0.4% NaCl. The absorption spectra were registered before and immediately after the illumination, in a Shimadzu UV-2100 spectrometer, to quantify the amount of I_3_^−^ generated in the different media, following published procedures (18).

### Confocal microscopy analysis of photosensitizer permeation in biofilms

Bacterial colonies from a fresh overnight plate were resuspended in BHI NaCl or BHI glucose and adjusted to OD_600_ = 0.02, and biofilms were grown in 8-well chamber slides for 24 h. The supernatant was then aspirated and replaced with fresh medium before the biofilm was exposed to 1 µM IC-H-Me^2+^ for 1 h. Next, the supernatant from each chamber was carefully removed, and the biofilm was washed twice gently with sterile saline. SYTO-9 solution (2.5 µM) was then added into each well and incubated at room temperature for 10 min while shielded from light before the staining solution was aspirated and the biofilm was washed again with sterile saline. The biofilms were fixed with formalin (3.7% PFA) at room temperature for 30 min, washed twice with saline, and a mounting medium and cover slip were applied. Biofilms were visualized and analyzed using a Zeiss 880 confocal microscope and Zeiss ZEN software. SYTO-9 was excited at 488 nm, and its fluorescence was observed at 500–540 nm. IC-H-Me^2+^ was excited at 405 nm, and its fluorescence was observed at 660–760 nm.

### Live/dead stain after aPDT treatment by confocal microscopy

Biofilms grown in 8-well chamber slides were incubated with 1 µM IC-H-Me^2+^ and 50 mM KI or PBS (control) for 1 h in the dark at room temperature before being irradiated with 660 nm LED with an LD of 5 J cm^−2^. Thereafter, the supernatants from each chamber were carefully removed, and biofilms were washed gently twice with saline before SYTO-9 solution (2.5 µM) and propidium iodide (15 µM) were added and incubated at room temperature for 10 min while shielded from light. Fluorescence microscopy was performed as described above. PI was excited at 561 nm, and its fluorescence was observed at 500–540 nm.

### Statistical analysis

A log transformation of bacterial CFU data was performed prior to statistical analyses to approach a normal distribution. The data are presented as mean ± SD of three experiments unless otherwise stated. Statistical significance was assessed using one-way analysis of variance (ANOVA) in GraphPad Prism and indicated as *, *P* ≤ 0.05; **, *P* ≤ 0.01; ***, *P* ≤ 0.001.
